# Home culture and its effects on English as a *lingua franca* communication: Voices from Chinese students at a United Kingdom university

**DOI:** 10.3389/fpsyg.2023.1057315

**Published:** 2023-03-10

**Authors:** Xiao Zhang, Christiane Lütge

**Affiliations:** Faculty of Foreign Language and Literature Studies, Ludwig-Maximilians-Universität München, Munich, Germany

**Keywords:** ELF, home culture, Chinese international students, intercultural communication, ELT

## Abstract

In academic research on intercultural communication (IC), students’ perceptions and experiences regarding English as a *lingua franca* (ELF) have been central to the discussion because they form the basis for English teaching policies and practices in multilingual and multicultural environments. Substantial theoretical research on ELF has called for a paradigm shift from emphasizing the over-simplistic correlation between language and Anglophone cultures to recognizing the legitimacy of non-native English learners’ home culture in English teaching pedagogy. Nonetheless, little empirical research has been conducted to examine how ELF speakers understand their home culture in ELF communications. Relatively fewer studies have investigated to what extent ELF users’ perceptions of home culture influence their IC practices. To address these gaps, this study aims to explore Chinese international students in a liberal arts university in the United Kingdom and their understanding of Chinese culture in authentic ELF interactions. In addition, the perceived effects of Chinese culture on students’ IC were explored in great depth. This study adopts a mixed-method approach, including a student questionnaire (*N* = 200) and follow-up semi-structured interviews (*N* = 10). Following descriptive statistics and thematic analysis of the obtained data, the findings revealed that most participants lacked a thorough understanding of their home culture, while they considered home culture playing a significant role in ELF communications. The contribution of this study builds on work in English users’ awareness of home culture in IC to identify the significance of enabling the presence of English learners’ home culture in English language teaching (ELT) classrooms.

## 1. Introduction

Intercultural communication (IC) scholarship is grounded in multiple disciplinary research areas, such as psychology, communication studies, linguistics, anthropology, and cultural studies, with a practical interest in understanding how people from different cultures could communicate more effectively (McLuhan, 1962). Focusing on discourse structures in IC, linguistic scholars (e.g., Lado, 1957; Ten Thije, 2003; Sharifian and Jamarani, 2013) inquired into the formation of discourse and examined to what extent different linguistic means could contribute to intercultural understanding. For example, discourse analysis has offered a methodological framework for Scollon and Scollon (2001), who conceptualize IC as ‘interdiscourse communication’, including the entire range of communications across social, ethnic, generational and gender boundaries. Considering the ubiquity of cultural and language contacts in intercultural encounters, applied linguists (e.g., Jiang, 2006; Liddicoat, 2009; Kramsch, 2013; Jackson, 2014; Zhu, 2014; Baker, 2015a; Baker and Sangiamchit, 2019) emphasized the interactional and linguistic aspects of IC that take place between people from various linguacultural backgrounds, in most cases using a second language. Following this, this study adopts Jackson’s (2014) definition of IC, which stands for ‘interpersonal communication between individuals or groups who are affiliated with different cultural groups and/or have been socialized in different cultural (and, in most cases, linguistic) environments’ (p. 3). Such an anti-essentialist view emphasizes culture as flexible and contextualized discourse patterns that go beyond the national level and entail a dynamic interpersonal dimension of communication. By this definition, IC is not merely an information exchange process across cultures but involves an interactive and fluid mediation process in which interlocutors’ cultures are negotiated and brought into contact (Baker, 2011). As such, correlating a language and a culture, such as English and United States, is unjustifiable when Italian students communicate with German professors in English.

Given that the majority of IC take place in English around the world (Wolf, 2015), English has been evidently recognized as a *Lingua Franca* (ELF) between people whose first language is not English. Currently, the overwhelming majority of English users are non-native English speakers, fostering a shared ownership of English among all users instead of merely the property of Anglophone countries (Seidlhofer, 2011; Widdowson, 2017; Galloway and Rose, 2018). The sense of global ownership of English has fundamentally changed the traditional goals of English language teaching (ELT) and questioned the exclusive norm Anglophone cultures serve in ELT classrooms (e.g., McKay, 2002; Jenkins, 2006; Kirkpatrick, 2012; Baker, 2015b). ELT pedagogy should be re-formulated by breaking the dependency on Anglophone cultures and empowering L2 (foreign/s language) English learners by engaging them with diverse cultures as legitimate bilingual or multilingual speakers (Jenkins, 2006; Pakir, 2009). In today’s globalized society, L2 English learners are expected to be able to communicate with culturally diverse people (Baker, 2011; House, 2012; Baker, 2015a,b). With that said, exposing learners to Anglophone cultures cannot meet up with their current communication needs in multilingual and multicultural environments (Cameron and Galloway, 2019). Instead, the cultural repertoire of L2 English learners’ home culture should be acknowledged in ELT as an effort to claim learners’ ownership of English (McKay, 2003a; House, 2012; Kumaravadivelu, 2012).

As a response to ELF, applied linguists (e.g., McKay, 2002; Jenkins, 2006; Baker, 2009, p. 574; Kirkpatrick, 2012) call for extending cultural contents to encompass English language learners’ home culture, or as Cortazzi and Jin (1996) termed the ‘source culture’ at the individual, discursive, and community levels. From the sociocultural perspective, McKay (2003a) states that ELT teachers should incorporate topics that deal with L2 English learners’ own cultures and select appropriate methodologies informed by the local context. Matsuda (2012) pinpoints that ELT should ‘prepare learners for the linguistically and culturally diverse world and represent English as a pluralistic and dynamic entity rather than a monolithic and static one’ (p. 169). That is, there is no fixed association between ELT and specific cultures. Instead, English learners’ home culture involving a complexity of changing beliefs, values and thoughts should be acknowledged.

In recent years, intercultural education has become a primary focus in China’s ELT, as the ‘One Belt, One Road’ initiative is gaining momentum (Simpson et al., 2022). Ministry of Education, People’s Republic of China (2020) has revised the national ELT curriculum, expounding that one fundamental objective is to strengthen Chinese students’ confidence in Chinese culture, enable them to describe Chinese culture in English, and increase their intercultural sensitivity. In such a context, Chinese L2 English learners’ perceptions of home culture have garnered academic attention (e.g., Song and Xiao, 2009; Xiao et al., 2010; Liu and Fang, 2017; Liu et al., 2018). For example, Liu and Fang’s (2017) study on English learners in China found that most of their understanding of Chinese culture (e.g., beliefs, values, and communication) was rather superficial, while they reported critical roles of awareness of Chinese culture in negotiating with culturally diverse people in IC. This finding was congruent with the study of Liu et al. (2018), who showed that 150 English-major students were positive about having more opportunities to become exposed to Chinese traditional culture (e.g., acupuncture, paper-cutting, and analects of Confucius). However, the participating students reported that English instructors seemed hesitant to integrate Chinese culture into their ELT classrooms due to the lack of teaching materials, teacher training and practical guidance.

While previous studies have highlighted that cultural globalization requires L2 English learners to gain exposure to various cultures other than those of English-speaking countries to communicate with people from different linguacultural backgrounds successfully, what is missing is an exploration of how English users navigate between their home culture and other cultures in realistic IC. Therefore, this study intends to investigate Chinese international students’ perceptions and awareness of home culture and further examine how their perceptions of home culture influence IC processes. The findings of the present study will provide valuable insights for English language instruction through which learners can increase their appreciation of their own cultures and explore their cultural identities in multicultural contexts (Kumaravadivelu, 2012; Sung, 2014, 2020; Baker, 2015a).

## 2. Literature review

### 2.1. Teaching culture in ELT

Culture is essential in IC as it not merely includes a set of shared beliefs, values, and concepts but molds individuals’ patterns of communicative behaviors (Tang, 2006; Holliday et al., 2021). Considering the global status quo of English, this study adopts the critical postmodernist stance of culture, which moves beyond the sum of a nation’s factual knowledge; instead, it should be conceptualized as dynamic, complex, and negotiable (Baker, 2008; Sharifian, 2017). Such a view considers culture as a fluid social practice that is individual-and contextual-dependent among speakers of discourse communities (Michelson, 2018). Home culture in this study stands for ‘Chinese culture’, which is understood as dynamic and multifaceted. It should be noted that while it is necessary not to take an essentialist view of culture, extensive studies (e.g., Holmes, 2006; Shi, 2006; Zhou and Todman, 2008; Gao, 2010; Gu, 2010; Mao and Qian, 2015; Liu and Fang, 2017) have summarized characteristics of communication manifested in Chinese culture, including face-saving, conforming with the vast majority, being shy to express opinions and avoiding negative comments. These major Confucian nurtured cultural traits different from others, especially Western cultures, may hinder Chinese students from participating in successful IC (Heng, 2017; God and Zhang, 2018a,b).

English language teaching has emphasized the inherent relationship between culture and language (Kramsch, 1993, 2009; Corbett, 2003; Risager, 2007), and the ideology of native-speakerism has dominated for decades. Nonetheless, the extent of English use on a global scale and the concept of culture as a dynamic and relational entity challenges the dominance of Anglophone cultures. From the perspective of ELF, learners should be exposed to various cultures for successful participation in IC (Baker, 2011, 2015a; Liu and Fang, 2017). Accordingly, Kramsch (2006) argues that learners should develop a cultural position from which they can mediate between their culture and the target language. Therefore, the culture teaching paradigm should move beyond ‘taxonomies of national culture features’ towards ‘cross-cultural understanding in the concrete situations of everyday life’ (Kramsch, 2002, p. 10).

The separation between English learning and Anglocentric culture has extended to incorporate learners’ home culture into the cultural teaching paradigm of ELT (Fairclough, 1995; McKay, 2003b; Canagarajah, 2005; Liu and Fang, 2017). As Canagarajah (2005) contended, English as a dominant language worldwide is forcing an unfamiliar pedagogical and social culture on non-native English learners, whose home cultures are devalued. Additionally, considering the key role of ELF materials and culture in English language acquisition, a body of research (e.g.,^51,52^; Vettorel, 2018; Si, 2020; Toledo-Sandoval, 2020; Guerra et al., 2022) explored representations of different cultures in produced coursebooks, while results present an orientation towards native-speaker standard English and related cultures with limited exposure of local cultures. A pluralistic, multicultural perspective in teaching materials is not given sufficient attention. Therefore, the locally-informed pedagogy shall integrate learners’ home culture into teaching practices and foster learners’ awareness about the ownership of English.

### 2.2. Interactions between Chinese English learners’ home culture and IC

Since English language speakers’ home culture plays a prominent role in the mediation process of ELF communications (Liu and Fang, 2017), applied linguists in China have reiterated the significance of home culture in challenging linguistic imperialism. For instance, Guo and Beckett (2007) lamented that most Chinese learners believed in the superiority of Anglo-culture and the inferiority of their own culture, which led to the neglect of learners’ knowledge about Chinese culture. Localized ELT curriculum and teaching materials are expected to manifest Chinese learners’ culture. Wang (2015a) found out that English teachers in China followed native English norms, which therefore constrained Chinese learners’ English language choices. Further, she suggests that educational practitioners explore various ways toward ELF-relevant content in classrooms. Similarly, Gu (2016) and Liu and Fang (2017) argued that Chinese learners should develop intercultural awareness and enable themselves to reflect on their own cultures in the English acquisition process. Additionally, a wide range of studies (e.g., Wen, 2012a; Wang, 2015a,b; Rai and Deng, 2016; Jin and Cortazzi, 2017) delved into the Chinese ELT context and emphasized the insufficiency of presenting target language cultures. To address the native speaker myth, they encourage Chinese learners to develop multicultural awareness, knowledge and understanding of different intercultural circumstances for ELF communications.

Apart from China’s domestic calls for integrating L2 English learners’ own culture into ELT classrooms, plenty of research (e.g., Rawlings and Sue, 2013; Zhang, 2016; God and Zhang, 2018a,b; Zhang et al., 2021) made attempts to understand Chinese international students’ IC experience through the lens of Confucian heritage culture (CHC). Influenced by CHC, which advocates endurance, conservation, respectful listening, and harmony over questioning (Zhang et al., 2021), Chinese international students were passive about and disengaged in IC. For example, Holmes (2005, 2006) explored communication difficulties encountered by Chinese students in a New Zealand university. She found that Chinese students’ communication styles and practices seemed not consistent with local standards in New Zealand. Likewise, God and Zhang (2018b) revealed that Chinese students could not appropriately communicate with local peers because of the collectivism–individualism divergence between Chinese and Australians.

Nevertheless, two significant gaps remain unfilled. First, given the necessity of English language speakers’ home culture in establishing interculturality in ELT in Chinese higher education, a paucity of research has been conducted to examine how Chinese students understand their own culture at practical levels. Second, although extensive research has focused on Chinese international students’ IC experiences, little is known about how their home culture affects IC. As Wu (2015) argued, an understanding of how Chinese culture influences their students’ outlook and behavior is essential for the development of intercultural education. As such, this study aims to explore Chinese international students’ perceptions of home culture and the perceived effects of home culture on their English-mediated communications in the United Kingdom. Two research questions are formulated:

1. How do Chinese international students perceive home culture in ELF communications?
2. To what extent do Chinese international students’ perceptions of home culture influence their ELF communications?

## 3. Methodology

### 3.1. Context and participants

This study took place in a small-scale liberal arts university in the UK. The majority of disciplines offered by this university are about social and human sciences. At the time of data collection, 12% of the students were international students (primarily females) from 23 different nationalities. English serves as the default communication means of IC among students from various linguacultural backgrounds.

A total of 200 Chinese students (Female = 128, Male = 72) were involved based on a convenient sampling, which is one of the non-random sampling strategies. The study sampling began with a convenient sample of 20 Chinese students who referred the researchers to other participants in the same university. The participants’ ages ranged from 19 to 32, and the length of residence abroad was from 6 months to 11 years. A percentage of them (31.5%) were pursuing Bachelor’s degrees, and the rest were master’s students. The average of participants’ English learning experiences is 10 years. They speak Mandarin as their first language and resort to ELF in intercultural interactions. Afterwards, ten participants participated in the semi-structured interviews, and their profiles are listed in Table 1.

**Table 1:** The profile of interviewees.

Name	Major	Gender	Length of residence
S1	Medicine	Female	5 years
S2	British literature	Female	6 months
S3	International education and global development	Female	7 months
S4	Electronic information	Male	11 years
S5	Intercultural communication	Female	4 years
S6	Finance	Female	18 months
S7	Sport and wellness management	Male	15 months
S8	Logistics	Female	5 years
S9	Digital media and culture	Female	6 months
S10	Business administration	Male	8 years

### 3.2. Data collection

This study employed a mixed-method approach which allows authors to collect data about the broader societal and individual context and avoid over-simplistic and superficial characterizations (Dörnyei, 2007, p. 45). It included two phases. First, a Chinese version questionnaire was adapted from Liu and Fang (2017) to obtain an overview of participants’ awareness of home culture. Second, semi-structured interviews with ten Chinese students were conducted to explore participants’ experiences and preferences from emerging patterns of the survey results (Kvale and Brinkmann, 2009). Interview attendants were informed of the study purpose in each interview, guided by probing questions. Each interview lasted around 40–50 min. To ensure clear responses to interview questions without language barriers, all interviews were conducted in Mandarin.

Before the data collection, a pilot study was conducted with 30 Chinese international students to test the reliability and clarity of questionnaire items. At the same time, two applied linguists in ELT were invited to participate in the survey finalization. Based on the feedback, minor modifications were made, such as changing the wording of the question from ‘what factors make influence when you talk about Chinese culture in English during intercultural communication?’ to ‘what impedes you from talking about Chinese culture in English in intercultural communication?’. All questions were closed-ended and administered anonymously to guarantee anonymity.

### 3.3. Data analysis

Quantitative data were fed into SPSS 24, and descriptive statistics, including frequency analysis, were adopted to summarize patterns of participants’ responses. In terms of the qualitative analysis, all interviews were recorded, transcribed, and translated verbatim. Transcripts were analyzed *via* Nvivo 10 for coding purposes through thematic analysis, focusing on meaning condensation and synopsis of meanings by participants into shorter formulations (Kvale and Brinkmann, 2009).

## 4. Findings

### 4.1. Questionnaire results

Questions 1–5 were formulated to answer the first research question. According to Table 2, participants expressed a positive attitude toward the importance of Chinese culture in IC, with 99 (49.5%) students somewhat agreeing and 72 (36%) students strongly agreeing with the statement. Regarding participants’ knowledge of Chinese culture (see Table 3), the majority (*N* = 136, 68%) reported a general understanding, while a small part of them (*N* = 63, 31.5%) articulated a thorough understanding of home culture.

**Table 2:** Participants’ perceptions of the importance of Chinese culture in intercultural communication (IC).

Knowing Chinese culture is important in intercultural communication		
Options	Frequency	Percent
Strongly disagree	9	4.5
Somewhat disagree	20	10
Somewhat agree	99	49.5
Strongly agree	72	36

**Table 3:** Participants’ knowledge of Chinese culture.

To what degree do you describe your knowledge and understanding of Chinese culture?		
Options	Frequency	Percent
I have a complete understanding.	63	31.5
I have a general understanding.	136	68
I know several parts of Chinese culture.	1	0.5
I have no idea about Chinese culture at all.	0	0

Concerning the participants’ willingness to introduce Chinese culture in IC (see Table 4), 87 (43.5%) participants reported a very willing attitude, and 80 (40%) participants expressed that they were willing to do so. For question 4, addressing the importance of Chinese cultural awareness in IC, most participants (*N* = 107, 53.5%) chose ‘very important’; only 7 (3.5%) expressed an indifferent attitude.

**Table 4:** Participants’ willingness to introduce Chinese culture.

Are you willing to introduce Chinese culture to people from other cultures when you talk to them?		
Options	Frequency	Percent
Very willing	87	43.5
Willing	80	40
Indifferent	32	16
Not very willing	1	0.5

Question 5 touched upon different topics relevant to Chinese culture that participants have discussed in their ELF communications. The results showed that Chinese food, tradition, festivals, customs, and history were most frequently discussed. Four participants supplemented Chinese relevant fashion trends, lifestyle, languages, traditional sports, and superstitions. Those responses imply that a variety of Chinese cultural aspects have been mentioned during Chinese students’ interactions with other international groups, while approximately 90% of participants placed importance on daily life experiences.

Questions 6–8 addressed how participants’ perceptions of Chinese culture influenced their intercultural interactions. Table 5 showed that half of the participants (*N* = 100, 50%) indicated that Chinese culture is to some degree facilitative for understanding other cultures. Nonetheless, a tiny portion (*N* = 7, 3.5%) thought that the knowledge of Chinese culture constrains their IC practices. The answers to question 7 further confirmed the significance of understanding Chinese culture in IC. 97 (48.5%) of the participants believed that, to some degree, an understanding of Chinese culture helps them communicate in intercultural settings. These results demonstrate that participants have acknowledged the significance of home culture in IC.

**Table 5:** Participants’ perceptions of Chinese culture toward understanding other cultures.

How do your knowledge and understanding of Chinese culture affect your understanding of other cultures		
Options	Frequency	Percent
It greatly helps me	55	27.5
It, to some degree, helps me	100	50
It does not affect my understanding	37	18.5
It, to some degree, limits my understanding	7	3.5
It greatly limits my understanding	1	0.5

In Question 8, participants voiced significant factors that hinder them from communicating Chinese culture in ELF communications. Of these responses, limited English language skills (*N* = 147, 73.5%) and a lack of knowledge about Chinese culture (35%) were perceived as two prominent obstacles. Besides, a portion of students admitted that the lack of reflection on Chinese culture (*N* = 59, 29.5%), insufficient resources (*N* = 31, 15.5%), weak interests (*N* = 24, 12%) and a lack of confidence about Chinese culture (*N* = 15, 7.5%) inhibited them from addressing Chinese culture with other international communities.

### 4.2. Qualitative findings

This section presents findings from the thematic analysis approach by concentrating first on limited knowledge and understanding of home culture to Chinese culture help to build common grounds and adopt critical perspectives and then moving to provide home cultural knowledge to bridge gaps. Pseudonyms are employed.

#### 4.2.1. Limited knowledge and understanding of Chinese culture

Before examining the perceived effects of home culture in IC, participants’ knowledge of home culture was investigated. Eight students were relatively confident about a part of Chinese cultural aspects, such as food, history, and philosophy. S10 claimed that:

S10: I know some about Chinese history and philosophy because I have read many books. […] Nevertheless, I only acquired limited knowledge about other Chinese cultural aspects when I was a student in China.

Additionally, two participants indicated that they had only surface knowledge and a superficial understanding of home culture while they expressed their interests. As S9 mentioned: ‘I only have a limited understanding of Chinese culture without much professional training, but I am interested in it’.

When discussing the awareness and knowledge of Chinese culture, S2 admitted the lack of a comprehensive understanding of Chinese culture but referred to several internationally famous Chinese celebrities and English versions of the remake of Chinese series. These responses may suggest the imbalance towards Anglophone cultures in ELT classrooms. This offers Chinese students little opportunities and space to reflect on their own cultures (Kumaravadivelu, 2012; Baker, 2015a).

#### 4.2.2. Home culture helps to build common grounds and adopt critical perspectives

Although Chinese international students’ understanding of home culture is limited, they have recognized the indispensability of home cultural traditions in approaching other cultural elements. Some participants voiced that they applied the comparative approach to analyze and compare different cultures with their own cultures to discover similarities. Eventually, this approach allows participants to recognize common grounds between cultures in specific situations and work as personal agents of their own changes (Kettle, 2005; Hu and Dai, 2021). S4’s intercultural practices were typical:

S4: My Chinese cultural background can help me. There are overlaps between different cultures. I think that everyone in the world is alike. There is a 90% chance that what happens in China might take place in other countries. I expanded my intercultural horizons.

In a similar vein, S9 believed in common beliefs between the Chinese and other cultures. She discussed similarities between various religions based on her experiences. These examples demonstrate that some participants show the propensity to minimize cultural differences (Bennett, 1993). A part of the participants embraced the belief that cross-cultural similarities were noticeable, with differences acknowledged but minimized.

It is worth highlighting that apart from the similarity of cultures mentioned above, cultural differences between their own and other cultures may enhance participants’ inclusive attitudes and cross-cultural awareness. These perceptions encouraged them to recognize different cultures and reconstruct their identities in the IC process. As Dai (2013) and Shen (2019) explained, the construction of intercultural identity is a process of cultural acceptance and establishing harmonious intercultural relations based on home culture by interpreting different cultural elements of the new culture instead of entirely abandoning either culture. S3, S6 and S5 shared their thoughts accordingly:

S3: Well, I like comparing the differences between my culture and other cultures, and I can deeply understand different cultures in the comparative process.

S6: I generally use my own culture to think about why people from other cultures believe that way. Using my culture helps me to perceive diversity in the world. Differences derive from different cultures.

S10: I make a horizontal comparison between China's history and that of other countries. The comparative approach helps me to understand different cultures. For example, what happened in the UK when China entered the Tang Dynasty made me better understand British and Chinese histories.

These responses suggest that participants realized differences between their own cultural beliefs and those of the host country, but they preferred to establish relationships between their own culture and other cultures. Meanwhile, this reflects participants’ intercultural awareness, which manifests in the ability to compare different cultures (Baker, 2011).

#### 4.2.3. Providing home cultural knowledge to bridge gaps

When participants socialized with peers from other cultures, several students reported that it was a positive start to initiate conversations if they shared some Chinese cultural traditions with other international students. In many ELF communication circumstances, the participants anticipated that some cultural topics and trends were unique to their home culture. As a result, they elaborated on local cultural items to bridge knowledge gaps in their recipients and open a dialog. Here are two examples:

S9: I tell friends from different cultures about the popular culture in China. This might not happen in other cultures. I have a lot to say when Chinese popular culture is involved in our conversations.

S2: I talked about my family with foreign friends, and I also explained how the Chinese family system developed. […] My cultural background and understanding of my country help me communicate with others. If I do not understand my culture, it is impossible to exchange information with people from other cultures.

These above extracts indicate that, on the one hand, students acquire new information and knowledge about new cultures; on the other hand, they actively introduce Chinese culture to engage in intercultural interactions. These experiences demonstrate that many participants acknowledged their Chinese cultural values and tended to highlight their home cultural topics in intercultural activities.

Nonetheless, it is worth noting that cultural heterogeneity between Chinese culture and other cultures leads to some barriers, which exert negative effects on participants’ communicative competencies and social practices. According to Holmes (2006) and Shi (2006), compared with Western cultures, Chinese culture is primarily influenced by Confucian values, which feature protection of the face and quiet rather than independent thinking. Thus, the misalignment between the Chinese collectivist notion and Western individualist cultures prevents Chinese students from fitting in with Western cultures. As S1 complained: ‘I think the Chinese way of learning and teaching cannot help me with being open-minded to adjust to new cultures. Similarly, S9 lamented that traditional Chinese cultural values hamper her IC skills: ‘Chinese culture teaches me to be conservative, face-saving, and humble. These values negatively impact my daily communication with western people.

These extracts demonstrate that cultural gaps between Chinese and other cultural characteristics are not conducive for Chinese students to reconstruct suitable communication styles in a new cultural context. These findings confirm Holmes’s (2006) opinion that Chinese international students encounter communication challenges in plurilingual social activities and should critically evaluate effective and appropriate communication strategies.

## 5. Discussion

This study examines Chinese international students’ perceptions of home culture and its effects on their ELF communications in a United Kingdom university. It compensates for the limitation of Liu and Fang (2017), who investigated Chinese domestic students without realistic intercultural experience. Notably, the majority of participants had an insufficient understanding of home culture, and most of them could mention only superficial aspects of home culture in IC processes. Two primary reasons derived from participants’ limited English language skills and lack of knowledge about hidden values and beliefs underneath Chinese culture. These findings confirmed previous studies (e.g., Shi, 2013; Zhou and Chen, 2015; Liu and Fang, 2017), where Chinese university students could not fluently communicate abstract aspects of Chinese culture in ELF. With that said, improving Chinese students’ English language skills is an urgent task for educational stakeholders in response to the ELT curriculum requirements of China.

Echoing the study of Sung (2022), who noted that international students in Hong Kong asserted their national identity and made an agentive approach to introduce their own culture to local peers, participants in this study saw their home culture as influential in the face of intercultural differences or even conflicts. The construction of a new cultural identity through recognizing home culture was present in the intercultural adaption process. By constantly comparing and reflecting on cultural similarities and differences, participants appeared more open-minded and inclusive towards diverse cultures. The participants were encouraged to develop their intercultural awareness and reshape their cultural identity in intercultural contexts by adding various cultural elements to their home culture. Finally, they strived to achieve a balance between their home culture and other cultures (Dai, 2012).

Furthermore, this study indicated that the majority of participants developed intercultural communicative competence and could integrate different views into their home cultures. In tune with Sung’s (2019, 2021) claim about the inextricable relationship between the role of personal agency and identity reconstruction, the findings suggest that the participants exercised their agency to build multiple intercultural identities grounded in their home culture. Nonetheless, these findings were divergent from Liu and Fang (2017), who concluded that Chinese students were obsessed with Western cultures without critically reflecting on their own culture. The reason might be that participants in their study did not have authentic abroad experience and received a monolithic Chinese way of ELT. Thus, it seems logical to conclude that ELT in China departs from the global outlook in the classroom and that Chinese students are unprepared for their English-medium encounters outside the classroom (Wang, 2015b).

## 6. Implications

The findings of this study provide several implications for the inclusion of home culture in ELT. First, L2 English learners’ English proficiency requires educators’ attention because it is the most significant impediment to smooth IC. For that reason, it is necessary for teachers to assess students’ English levels and design a teaching curriculum underlying intercultural education.

Second, cultural teaching practices in ELT are another essential factor in overcoming IC barriers. On the one hand, teaching materials should be re-designed to incorporate students’ home cultures and break down the focus on Anglophone cultural representations (McKay, 2000, 2003a, b; Kumaravadivelu, 2012; Matsuda, 2012). On the other hand, instead of transmitting factual knowledge, L2 English learners’ home culture should be presented from an intercultural perspective in the ELT classroom, where students can develop their critical cultural awareness (Byram, 2008). As such, it will help students engage in their home culture and self-reflection in internationalized contexts. For instance, teachers can play movie clips about the case of miscommunication in intercultural settings and encourage students to reflect on clarifications of misunderstanding.

Third, it is advisable to encourage L2 English learner autonomy underpinning their highly individualized needs in the actual use of English. Instead of providing learners with obsolete teaching content, institutional and university administrators should supplement intercultural practices into the curriculum to provide learners with more communication opportunities with people from diverse cultures. Such teaching practices may assist domestic students and international groups in expanding cultural exchanges through ELF and building a mutual understanding of cultures, potentially forming a diverse intercultural learning context.

## 7. Conclusion

This study explored Chinese international students’ perceptions of home culture and the perceived role of home culture in ELF communications. Findings indicate that students’ understanding of home culture is fundamental, while they have been aware of the importance of home culture and its bearing on intercultural contacts. Notably, home culture is an integral part of Chinese international students’ intercultural awareness and influences their social practices in ELF communications. Students are likely to share their home culture and compare different cultures with their own in intercultural communities. In this way, they are enabled to become agents for re-negotiating different cultures. Therefore, this study justifies the inclusion of home culture into ELT and challenges native English cultural standards in culture teaching. However, it is worth mentioning that emphasizing home culture in ELT does not mean an ethnocentric view of culture; instead, this study supports an inclusive attitude by seeking cultural similarities and differences and a context-dependent cultural teaching approach. This study contributes to re-examining the relationship between the English language and culture teaching, encouraging teacher educators and practitioners to adopt a critical perspective to review the complexity of culture teaching.

Despite the contributions this study makes in terms of home culture in ELT, it has three limitations. Firstly, there is space for the questionnaire improvement regarding perceptions of students’ home culture. Future studies can incorporate more items for a comprehensive picture. Secondly, this study relies on a relatively small sample of participants; thus, the findings might not represent the whole population spectrum. Therefore, larger-in-scale participants with a numerical balance of male and female participants in future studies can be recruited for a broader range of voices. Lastly, the study shows that participants hold positive attitudes toward home culture and its positive effects on IC; it has yet to explore whether students’ attitudes correlate with some variables, such as gender, age, educational degree, or the length of residence abroad. Future research can investigate whether some independent variables correlate with students’ perceptions and awareness of their own culture.

## Data availability statement

The original contributions presented in the study are included in the article/supplementary material, further inquiries can be directed to the corresponding author.

## Ethics statement

The studies involving human participants were reviewed and approved by Ethics Committee of Foreign Languages and Literatures, Ludwig-Maximilians-Universität München. The patients/participants provided their written informed consent to participate in this study.

## Author contributions

XZ designed the study, collected the data, and wrote the draft. CL revised and finalized the manuscript. All authors contributed to the article and approved the submitted version.

## Conflict of interest

The authors declare that the research was conducted in the absence of any commercial or financial relationships that could be construed as a potential conflict of interest.

## Publisher’s note

All claims expressed in this article are solely those of the authors and do not necessarily represent those of their affiliated organizations, or those of the publisher, the editors and the reviewers. Any product that may be evaluated in this article, or claim that may be made by its manufacturer, is not guaranteed or endorsed by the publisher.
